# Decreased spinal cord motor neuron numbers in mice depleted of central nervous system copper

**DOI:** 10.1093/mtomcs/mfae036

**Published:** 2024-09-09

**Authors:** J R Liddell, J B W Hilton, Y J Wang, J L Billings, S Nikseresht, K Kysenius, J P Fuller-Jackson, D J Hare, P J Crouch

**Affiliations:** Department of Anatomy and Physiology, The University of Melbourne, Parkville, VIC 3010, Australia; Department of Anatomy and Physiology, The University of Melbourne, Parkville, VIC 3010, Australia; Department of Anatomy and Physiology, The University of Melbourne, Parkville, VIC 3010, Australia; Department of Anatomy and Physiology, The University of Melbourne, Parkville, VIC 3010, Australia; Department of Anatomy and Physiology, The University of Melbourne, Parkville, VIC 3010, Australia; Department of Anatomy and Physiology, The University of Melbourne, Parkville, VIC 3010, Australia; Department of Anatomy and Physiology, The University of Melbourne, Parkville, VIC 3010, Australia; Atomic Medicine Initiative, University of Technology Sydney, Sydney, NSW 2007, Australia; Department of Anatomy and Physiology, The University of Melbourne, Parkville, VIC 3010, Australia

**Keywords:** solute carrier family 31 member 1 (Slc31a1 Ctr1), copper, motor neuron, amyotrophic lateral sclerosis (ALS), central nervous system (CNS), mouse model

## Abstract

Disrupted copper availability in the central nervous system (CNS) is implicated as a significant feature of the neurodegenerative disease amyotrophic lateral sclerosis (ALS). Solute carrier family 31 member 1 (Slc31a1; Ctr1) governs copper uptake in mammalian cells and mutations affecting Slc31a1 are associated with severe neurological abnormalities. Here, we examined the impact of decreased CNS copper caused by ubiquitous heterozygosity for functional Slc31a1 on spinal cord motor neurons in *Slc31a1^+/^^−^* mice. Congruent with the CNS being relatively susceptible to disrupted copper availability, brain and spinal cord tissue from *Slc31a1^+/^^−^* mice contained significantly less copper than wild-type littermates, even though copper levels in other tissues were unaffected. *Slc31a1^+/^^−^* mice had less spinal cord α-motor neurons compared to wild-type littermates, but they did not develop any overt physical signs of motor impairment. By contrast, ALS model *SOD1^G37R^* mice had fewer α-motor neurons than control mice and exhibited clear signs of motor function impairment. With the expression of *Slc31a1* notwithstanding, spinal cord expression of genes related to copper handling revealed only minor differences between *Slc31a1^+/^^−^* and wild-type mice. This contrasted with *SOD1^G37R^* mice where changes in the expression of copper handling genes were pronounced. Similarly, the expression of genes related to toxic glial activation was unchanged in spinal cords from *Slc31a1^+/^^−^* mice but highly upregulated in *SOD1^G37R^* mice. Together, results from the *Slc31a1^+/^^−^* mice and *SOD1^G37R^* mice indicate that although depleted CNS copper has a significant impact on spinal cord motor neuron numbers, the manifestation of overt ALS-like motor impairment requires additional factors.

## Graphical Abstract

**Graphical Abstract:**
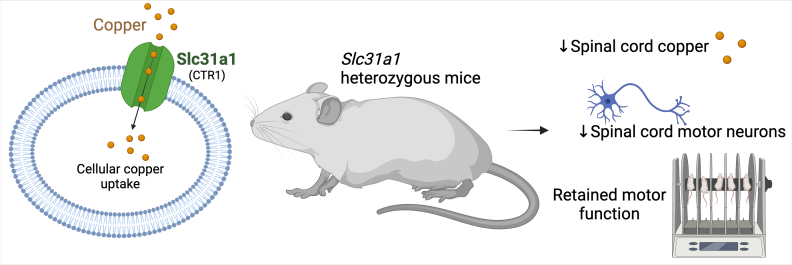
Decreasing central nervous system copper in mice affects spinal cord motor neuron numbers but does not affect motor function.

## Introduction

Amyotrophic lateral sclerosis (ALS) is the most common form of the neurodegenerative condition of motor neuron disease. It is characterized by the progressive demise of motor neurons in the brain and spinal cord, resulting in adult onset weakening and eventual paralysis of skeletal muscle throughout the body followed by premature death.^1^ Although there are >50 genes reported to be involved in ALS as either causal or increasing risk,^2^ the cellular and biochemical pathways to neuronal death in ALS still require elucidation. Additionally, the development of novel therapeutic interventions capable of modifying disease progression also requires further exploration.^3^

One therapeutic strategy that has gained attention is treatment of ALS by manipulating copper availability in the affected central nervous system (CNS). Copper is an essential micronutrient and the first discovered genetic cause of ALS was attributed to mutations affecting the ubiquitous and highly abundant antioxidant cuproenzyme superoxide dismutase 1 (SOD1).^4^ Transgenic mice expressing mutant *SOD1* have strong face and construct validity for ALS,^5^ and increasing spinal cord copper content in these models through transgenic overexpression of solute carrier family 31 member 1 (*Slc31a1*; Ctr1) or through treatment with the copper compound Cu^II^(atsm) is neuroprotective, mitigates the rate of decline of motor function impairment, and extends lifespan.^6,7^ Overexpressed SOD1 in mutant SOD1 transgenic mice is unsatiated for its stoichiometric requirement for copper in the CNS.^7,8^ By contrast, overexpressed SOD1 in peripheral tissues of the same mice (kidney, liver, and skeletal muscle) displays an increase in copper-dependent activity that is more commensurate with increased expression of the protein.^8^ Indicative of SOD1 not being the only cuproenzyme unsatiated for its requirement for copper in the CNS of mutant SOD1 mice, ceruloplasmin and hephaestin also appear unsatiated for their copper-dependent ferroxidase activity in CNS tissue from mutant SOD1 mice.^9^ Notably, these cuproenzymes appear to accumulate in a copper-deficient state in human cases of ALS.^10^ This includes sporadic cases of the disease with no known genetic history of the disease and is not known to be attributable to mutations affecting SOD1.^10^

These results indicate that disrupted availability of copper is a significant, CNS-specific feature of ALS and that modulating copper availability within the CNS may be a plausible therapeutic target for providing neuroprotection in ALS and improving motor function and survival. However, it is pertinent to note that although some cuproenzymes appear unsatiated for their physiological copper requirement in mutant SOD1 mice and human cases of ALS, total copper content of spinal cord tissue is not decreased, and some reports describe an overall increase in spinal cord copper.^9–15^ Furthermore, treatment with the copper ligating compound ammonium tetrathiomolybdate has also been reported to protect motor neurons and slow the rate of decline in motor function in ALS model mice.^14^ These reports on copper in ALS span sporadic human cases and numerous SOD1 animal models. Collectively, they indicate a complex role for spinal cord copper availability in motor neuron survival in ALS that is not restricted to specific SOD1 mutations and is yet to be fully elucidated.

The high-affinity copper transporter coded by *Slc31a1* (also known as copper transporter 1, Ctr1) is the principal transporter required for copper uptake in mammalian cells. Functional Slc31a1 is essential for development, with homozygous deletion of *Slc31a1* being embryonically lethal in mice.^16,17^ By contrast, mice heterozygous for functional Slc31a1 develop normally and have no obvious phenotype into middle adulthood.^16,17^ While copper levels in liver, kidney, and intestines are unchanged in these mice, copper levels in the brain are diminished by ∼50%.^16,17^ Thus, the brain appears to be more sensitive to *Slc31a1* heterozygosity than other organs. Selective sensitivity of the CNS to *Slc31a1* heterozygosity indicates that *Slc31a1^+/^^−^* mice can be utilized to model disrupted copper metabolism in the CNS without overt confounding factors introduced by peripheral effects.

While motor neuron survival in ALS appears to be impacted by the availability of copper, it is clear that the relationship between spinal cord copper content, motor neuron numbers, and motor function requires further examination. Herein, we examined these factors in mice that are heterozygous for functional endogenous Slc31a1.

## Materials and methods

### Mice

All experiments conducted with mice were approved by a small laboratory animal ethics committee (approval numbers 2015142 and 1814531) at the University of Melbourne and were conducted in accord with the Australian Code for the Care and Use of Animals for Scientific Purposes as well as relevant University of Melbourne Animal Use and Care Standards. Mice were kept under standard housing conditions. which included *ad libitum* access to chow and water and a 12-h light/dark cycle as well as cardboard boxes and rolls for enrichment and tissue paper for nesting.

Mice heterozygous for functional Slc31a1^17^ were purchased from the Jackson Laboratory (strain #025649) and a colony maintained by breeding with wild-type C57BL/6 J mice purchased from the Australian Animal Resources Centre (OzGene, C57BL/6JOzarc). Male and female *Slc31a1^+/^^−^* mice and their wild-type littermates were assessed for motor function and killed for tissue collection (as described in detail below) when they were between 9 and 11 months old.

ALS model mice with hemizygous expression of a transgene encoding human SOD1 with a glycine to arginine substitution mutation (*SOD1^G37R^*)^18^ were purchased from the Jackson Laboratory (strain #008342). These mice were assessed for motor function and killed for tissue collection (as described in detail below) when they were 6 months old. Assessing these mice at an older age is not feasible due to the level of physical impairment mandating humane, ethical intervention. Age- and sex-matched wild-type non-transgenic mice were used as controls. Both sexes were used in this study.

### Motor function assessment

Motor function was assessed using the rotarod assay based on procedures previously described.^9^ In brief, mice were habituated to the assay over a period of 5 days prior to recording motor function. Assessment of motor function involved recording latency to fail in six independent trials on the rotarod. For each trial, speed of the rotarod increased from 4 to 40 rpm linearly over a 180-s period. The average latency to fail was calculated across the six trials for each mouse.

### Tissue collection

Mice were killed for tissue collection as previously described.^9^ Briefly, the procedure involved deep anaesthetization using a cocktail of ketamine and xylazine, followed by transcardial perfusion using phosphate buffered saline supplemented with heparin, protease inhibitors, and phosphatase inhibitors. A small (∼5 mm) section of lumbar spinal cord was post-fixed in 4% (w/v) paraformaldehyde and then the remainder was snap frozen and stored at –80 °C. Quadriceps, liver, kidney, heart, and brain were also excised from the animals, snap frozen, and stored at –80 °C.

### Tissue copper content

Frozen tissue samples were homogenized in ice cold Tris-buffered saline (TBS) solution supplemented with protease and phosphatase inhibitors. TBS-soluble material was extracted by centrifuging (15 min, 4 °C, 21 000 RCF), and then collecting the supernatant. The remaining TBS-insoluble material was washed once by resuspending in the TBS homogenization buffer and then re-centrifuging and discarding the second supernatant. The remaining pellet was resuspended in the TBS homogenization buffer and designated TBS-insoluble material. This procedure results in enrichment of cytosolic material in the TBS-soluble fraction and enrichment of nuclear and plasma membrane components in the TBS-insoluble fraction.^19^ Aliquots of the TBS-soluble and TBS-insoluble samples were used to measure protein content using the BCA Protein Assay Kit (Pierce). Additional 0.5 μl aliquots were transferred to microscope slides, air dried in a contamination-free chamber, and then assessed for tissue copper content using the laser ablation inductively coupled plasma mass spectrometry ‘micro-droplet’ method previously described.^20^ Tissue copper content is expressed relative to protein content.

### Motor neuron counting

Fixed spinal cord tissue was embedded in paraffin and transverse sections were cut at 7 μm. An average of 17 sections was prepared per animal, with 43 μm of spinal cord omitted between each section. By applying this procedure, motor neurons from an ∼850 μm length of spinal cord were counted for each animal. Sections mounted on microscope slides were stained with neutral red, digitally scanned using a 3DHISTECH PANNORAMIC SCAN II scanner, and then visualized using CaseViewer. Motor neurons were counted as visually conspicuous cells lateral and ventral to the central canal, and α-motor neurons were designated as those with a soma diameter of 20 μm or more. Results are presented as the average number of motor neurons counted on both sides of the central canal for each animal.

### Gene expression

Uptake, partitioning, and utilization of copper in mammalian cells involves numerous transporters, chaperones, and cuproproteins.^21^ Frozen spinal cord samples were assessed for expression of genes related to copper uptake and partitioning, and also glial activation, using procedures previously described.^22^ In brief, RNA was extracted using TRI Reagent (Sigma), contaminating DNA degraded using the TURBO DNA-free Kit (Thermo Fisher Scientific), and cDNA was synthesized using the High Capacity cDNA Reverse Transcription Kit (Thermo Fisher Scientific). Then, 160 ng of cDNA was pre-amplified using TaqMan PreAmp Master Mix and pooled TaqMan Gene Expression Assays (Thermo Fisher Scientific) for 14 cycles. Quantitative reverse transcriptase polymerase chain reaction was performed on 20-fold diluted pre-amplified cDNA using TaqMan Gene Expression Assays and TaqMan Fast Advanced Mastermix on a QuantStudio 6 Flex system (Thermo Fisher Scientific). Relative gene expression was determined via the ΔΔ CT method normalized to *Gapdh* (Mm99999915_g1). TaqMan Gene Expression Assays used in this study for genes relating to copper handling were *Slc31a1* (Mm00558247_m1), *Atox1* (Mm00839626_m1), *Atp7a* (Mm00437663_m1), *Atp7b* (Mm00599675_m1), *Ccs* (Mm00444148_m1), *Commd1* (Mm01239669_m1), *Cox11* (Mm01615963_g1), *Cox17* (Mm01346225_m1), *Mt1* (Mm00496660_g1), *Mt2* (Mm00809556_s1), *Mt3* (Mm00496661_g1), *Mtf1* (Mm00485274_m1), *Mtf2* (Mm00489151_m1), *Sco1* (Mm01329074_m1), *Steap1* (Mm00459097_m1), *Steap2* (Mm01320129_m1), *Steap3* (Mm01287243_m1), *Steap4* (Mm00475405_m1), and *Xiap* (Mm00776505_m1). For glial activation, TaqMan Gene Expression Assays were used for the following genes: *Alox5* (Mm01182747_m1), *C1qa* (Mm00432142_m1), *C3* (Mm01232779_m1), *Cx3cr1* (Mm02620111_s1), *Gfap* (Mm01253033_m1), *Il1a* (Mm00439620_m1), *Lcn2* (Mm01324470_m1), and *Tnf* (Mm00443258_m1). All samples were assessed in triplicate per gene.

### SOD1 protein and activity

Levels of SOD1 and the enzyme's dismutase activity in TBS-soluble extracts of mouse spinal cord were both assessed as previously reported.^9,15^ In brief, SOD1 protein levels were determined using western blot and a primary antibody that detects both transgenic human SOD1 and the endogenous mouse protein (Abcam; ab16831). SOD1 protein levels were normalized relative to the loading control β-actin (Cell Signaling Technology; 8H10D10 #3700) and expressed relative to levels in wild-type/non-transgenic littermate control mice. Determination of SOD1 activity utilized the pyrogallol assay.^9,15,23^ Calculation of units of activity was based on cyanide-sensitive activity and reference to purified bovine SOD1 (Sigma–Aldrich; S7571).

### Lipid peroxidation

Assessment of lipid peroxidation was used to examine levels of oxidative stress in *Slc31a1^+/^^−^* mouse spinal cord samples utilizing a procedure previously described.^22^ In brief, frozen spinal cord samples (∼15 mg) were homogenized in the TBS-based homogenization buffer described above and then incubated with the lipid peroxidation sensor C11-BODIPY (Thermo Fisher Scientific; D3861). The ratio of oxidized to reduced C11-BODIPY was calculated and expressed relative to wild-type littermate controls. Lipid peroxidation data from SOD1^G37R^ mice (Fig. 5C) are from a previous report^22^ and reproduced under a Creative Commons Attribution 4.0 International License.

### Data analyses

All statistical analyses were performed using GraphPad Prism software. Statistical outliers were assessed using the ROUT method^24^ and data are presented as mean ± SEM. Significant differences between groups were determined using two-tailed *t*-tests or one-way ANOVA where multiple comparisons were corrected using Holm–Sidak's test. Significance was determined as *P* < 0.05.

## Results

**Fig. 1.**
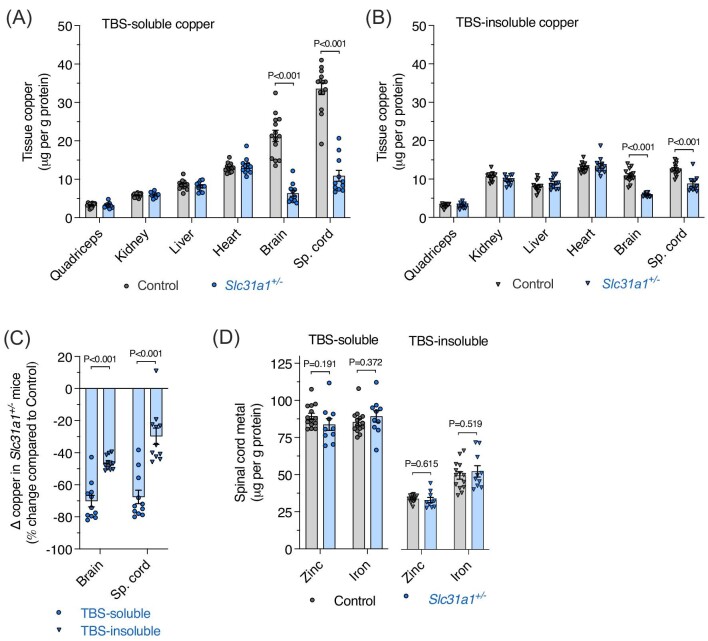
Copper content of tissues from *Slc31a1^+/^^−^* mice compared to wild-type controls. Tissue copper content is expressed as μg per g protein and presented for the TBS-soluble (A) and TBS-insoluble (B) fractions of the tissues shown. (C) Copper content of the TBS-soluble and TBS-insoluble extracts from *Slc31a1^+/^^−^* mice expressed as a percentage change relative to control mice. (D) Zinc and iron content in TBS-soluble and TBS-insoluble fractions of mouse spinal cord expressed as μg per g protein. Data are presented as mean ± SEM. Symbols represent values from individual mice, with *n* = 14 controls and *n* = 10–11 *Slc31a1^+/^^−^. P* values illustrate significant differences between the groups indicated.

Ubiquitous, heterozygosity for functional Slc31a1 in mice decreases total copper content of the brain by 50% compared to wild-type littermates while having no impact on total copper content in intestine, kidney, or liver.^16,17^ Our results support these original findings and additionally show a comparable impact on spinal cord copper (Fig. 1A and B). In both the brain and spinal cord, the TBS-soluble pool of copper was most affected in *Slc31a1^+/^^−^* mice when compared to the TBS-insoluble pool (Fig. 1C). In contrast to strong changes in spinal cord levels of copper in the TBS-soluble and TBS-insoluble fractions, neither iron nor zinc was affected (Fig. 1D), supporting copper specificity of the transporter and indicating that heterozygosity for functional Slc31a1 *in vivo* is not sufficient for induction of secondary effects involving related biometals.

**Fig. 2.**
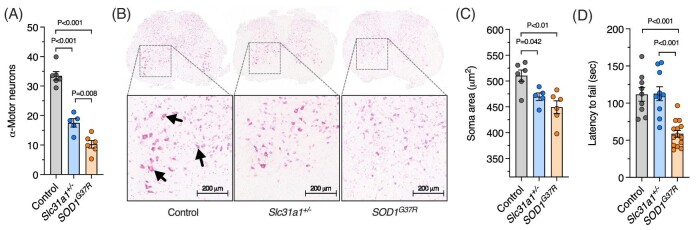
Spinal cord neurons in *Slc31a1^+/^^−^* mice compared to wild-type controls and ALS model *SOD1^G37R^* mice. (A, B) Average number of α-motor neurons per cross-sectional area in mouse spinal cords with accompanying representative histology images. (C) Average area of α-motor neuron soma in mouse spinal cords. (D) Motor function of mice assessed using rotarod assay and expressed as latency to fail. Data are presented as mean ± SEM. Circles represent values from individual mice, with *n* = 6 controls, *n* = 5 *Slc31a1^+/^^−^* and *n* = 6 SOD1^G37R^ in A and C, and *n* = 9 controls, *n* = 10 *Slc31a1^+/^^−^*, and *n* = 14 SOD1^G37R^ in D. *P* values illustrate significant differences between the groups indicated.

The TBS-soluble fraction contains cytosolic material, whereas the latter includes nuclear and plasma membrane components.^19^ Thus, in *Slc31a1^+/^^−^* mice, decreased copper levels were more pronounced in cytosol than in other cellular compartments. These large changes detected in spinal cord copper were associated with significantly fewer ventral α-motor neurons in *Slc31a1^+/^^−^* mice (Fig. 2A and B). Additionally, average soma area of the α-motor neurons present in the *Slc31a1^+/^^−^* mice was 9% smaller than in wild-type littermates (Fig. 2C). Despite these conspicuous and significant differences in α-motor neurons, *Slc31a1^+/^^−^* mice displayed no deficit on the rotarod assay for motor function relative to wild-type controls (Fig. 2D). This contrasted with ALS model *SOD1^G37R^* mice that displayed a 48% deficit on the rotarod assay. Notably, *SOD1^G37R^* mice had more pronounced decreases in α-motor neuron numbers and soma size when compared to *Slc31a1^+/^^−^* mice (Fig. 2A–C).

**Fig. 3.**
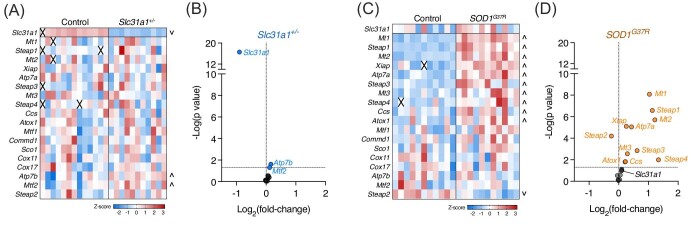
Expression of genes related to copper handling in mouse spinal cord extracts. (A, C) Z-score heatmaps illustrating gene expression differences in *Slc31a1^+/^^−^* mice compared to wild-type controls and *SOD1^G37R^* mice compared to non-transgenic controls. (B, D) Volcano plots showing fold-change differences for genes shown in A and C relative to statistical significance. Squares in A and C represent values for individual mice, with *n* = 13 controls and *n* = 11 *Slc31a1^+/^^−^* and crosses represent excluded samples. Circles in B and D represent mean fold-change values for individual genes. Arrow heads in A and C indicate significantly (*P* < 0.05) up- and downregulated genes in *Slc31a1^+/^^−^* and *SOD1^G37R^* mice relative to respective controls.

**Fig. 4.**
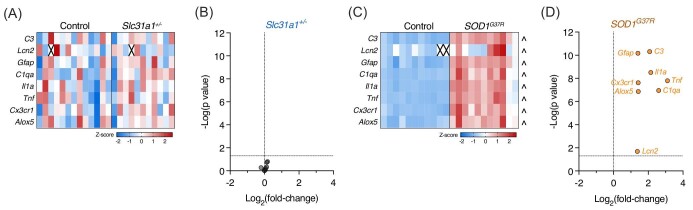
Expression of genes related to toxic glial activation in mouse spinal cord extracts. (A, C) Z-score heatmaps illustrating gene expression differences in *Slc31a1^+/^^−^* mice compared to wild-type controls and *SOD1^G37R^* mice compared to non-transgenic controls. (B, D) Volcano plots showing fold-change differences for genes shown in A and C relative to statistical significance. Squares in A and C represent values for individual mice, with *n* = 13 controls and *n* = 11 *Slc31a1^+/^^−^* in A, and *n* = 11 controls and *SOD1^G37R^* in C, and crosses represent excluded samples. Circles in B and D represent mean fold-change values for individual genes. Arrow heads in C indicate significantly (*P* < 0.05) upregulated genes in *SOD1^G37R^* mice relative to non-transgenic controls.

Subsequent to uptake by Slc31a1, cellular handling of copper and delivery to cuproenzymes involves a coordinated network of transporters and chaperones.^21^ Gene expression analysis of spinal cord extracts confirmed that *Slc31a1* transcript levels were decreased by ∼50% in *Slc31a1^+/^^−^* mice compared to wild-type controls (Fig. 3A and B). However, despite this decrease in *Slc31a1*, and despite the large resultant decrease in spinal cord copper levels (Fig. 1), expression levels for most of the other genes related to copper handling were unchanged in the *Slc31a1^±^* mice (Fig. 3A and B). Expression of *Atp7b* and *Mtf2* provided the only exceptions, being moderately elevated in the *Slc31a1^±^* mouse spinal cords at 12% (*P* = 0.024) and 8% (*P* = 0.049), respectively, above wild-type controls. By contrast, expression of 11 out of 19 copper-handling genes analysed was significantly changed in spinal cords of *SOD1^G37R^* mice compared to non-transgenic controls (Fig. 3C and D). Expression of *Slc31a1* was not changed in the *SOD1^G37R^* mice. Similar to results observed for expression of genes related to copper handling, the expression of genes related to toxic glial activation^25^ was highly upregulated in *SOD1^G37R^* mice but relatively unchanged in *Slc31a1^+/^^−^* mice (Fig. 4).

**Fig. 5.**
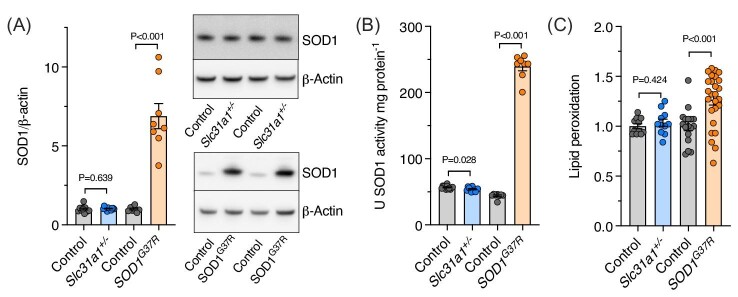
SOD1 and lipid peroxidation in mouse spinal cord extracts. (A) SOD1 protein levels in *Slc31a1^+/^^−^* mice compared to wild-type controls and *SOD1^G37R^* mice compared to non-transgenic controls. Representative western blots are shown. (B) Cyanide-sensitive SOD1 activity expressed as units of activity relative to a purified bovine SOD1 standard. (C) Lipid peroxidation levels in spinal cord homogenates determined using the ratiometric fluorophore C11-BODIPY and expressed relative to control mice. Data are presented as mean ± SEM. Circles represent values from individual mice, with *n* = 12 controls and *Slc31a1^+/^^−^* and *n* = 8 controls and SOD1^G37R^ in A and B, and *n* = 11 controls and *Slc31a1^+/^^−^* and *n* = 17 controls and 26 SOD1^G37R^ in C. *P* values illustrate significant differences between the groups indicated.

Consistent with the SOD1^G37R^ mouse model of ALS involving overexpression of a transgene,^18^ SOD1 protein levels were elevated in TBS-soluble extracts from spinal cords of these mice (Fig. 5A). By contrast, only endogenous SOD1 protein was present in spinal cord extracts from *Slc31a1^+/^^−^* mice. As the G37R mutation does not directly affect SOD1 activity, dismutase activity in spinal cord extracts from SOD1^G37R^ mice was significantly elevated compared to non-transgenic controls (Fig. 5B). SOD1 activity in spinal cord extracts from *Slc31a1^+/^^−^* mice was modestly (<5%) but significantly decreased. To associate these SOD1 related changes with oxidative stress, we assessed spinal cord homogenates for levels of lipid peroxidation as previously reported for SOD1^G37R^ mice.^22^ In contrast to the ALS model, elevated lipid peroxidation was not evident in the *Slc31a1^+/^^−^* mice (Fig. 5C).

## Discussion

Herein, we show that a decrease in spinal cord copper of ∼50% caused by heterozygosity for functional Slc31a1 in mice (Fig. 1) is associated with a near-proportional decrease in the number of spinal cord α-motor neurons (Fig. 2). Given that ubiquitous heterozygosity for functional Slc31a1 is from conception in *Slc31a1^+/^^−^* mice, it is not possible to determine from the current assessment of these animals whether the observed decrease in motor neurons reveals a requirement for copper in motor neuron development, a requirement for copper in survival of mature motor neurons, or, potentially, a combination of both these possibilities. Regardless, retained motor function in the rotarod assay (Fig. 2D) indicates that despite the clear deficit in motor neuron numbers, *Slc31a1^+/^^−^* mice were not overtly physically impaired. It is possible that relatively subtle physical impairment may have been detectable in other assessments of motor function and/or coordination such as the grip strength, foot fall, or balance beam assays.

The sustained motor function observed in *Slc31a1^+/^^−^* mice despite motor neuron loss could be a product of compensatory adaptations among premotor circuits. Directly innervating α-motor neurons are V0_c_ interneurons.^26^ These interneurons have large cholinergic inputs onto α-motor neurons, known as C-boutons, through which they modulate neuronal excitability and coordination in a task-dependent manner.^26,27^ In mutant SOD1 mice, genetic silencing of V0_c_ interneurons appears to both worsen^28^ and improve^29^ motor function during the early stage of ALS-like phenotype progression. These seemingly opposing results were obtained from different motor function assays (treadmill and rotarod, respectively), suggesting differential impact of V0_c_ interneurons depending on the motor circuit involved. Hence, any histological assessment of C-bouton density and size must be undertaken systematically, focusing on specific motor neuron pools relevant to motor function assays.^30^ Such analyses require inclusion in the initial study design and were thus not possible in the present study.

Oxidative stress is a commonly examined mechanism for its potential role in neurodegeneration in ALS. Mutations affecting the frontline antioxidant SOD1 were the first discovered genetic cause of ALS^4^ and oxidative stress is a prominent feature of the disease.^31^ However, not all cases of ALS involve SOD1 mutations and not all ALS-associated SOD1 mutations are associated with loss of dismutase function.^32^ However, because SOD1 requires copper for its catalytic antioxidant activity,^33^ we examined levels of endogenous SOD1 in spinal cord tissue from *Slc31a1^+/^^−^* mice, copper-dependent dismutase activity of the enzyme, and lipid peroxidation levels as an indicator of oxidative stress. The observed marginal (<5%) decrease in SOD1 activity in the *Slc31a1^+/^^−^* mice despite no change in SOD1 protein levels (Fig. 5A and B) indicates a loss of SOD1 activity potentially due to decreased spinal cord copper levels. However, the absence of any detectable change in lipid peroxidation (Fig. 5C) indicates that the small decrease in SOD1 activity observed in the *Slc31a1^+/^^−^* mice was not sufficient to induce any readily detectable oxidative stress consequence. This contrasts with the SOD1^G37R^ mice where, although overall SOD1 activity was increased due to transgenic overexpression, lipid peroxidation is elevated. These data indicate that sustained motor function in the *Slc31a1^+/^^−^* mice may be related to the absence of significant oxidative stress. Moreover, although significant levels of oxidative stress may be associated with manifestation of an ALS-like deterioration of motor function in mice, a restricted supply of copper to SOD1 and diminished SOD1 activity may not be a key feature. Perturbations affecting copper availability contributing to motor neuron death and diminished motor function in ALS may involve toxic events not restricted to SOD1 activity.

An additional consideration for sustained motor function observed in the *Slc31a1^+/^^−^* mice is the involvement of toxic glial activation in ALS. The conversion of microglia and astrocytes that are physiologically essential for healthy neuronal function into a pathologically activated state that negatively impacts motor neuron function and survival is well recognized as an important feature of ALS.^34^ Here, we show that in contrast to the ALS model *SOD1^G37R^* mice that show a gene expression signature for deleterious activation of microglia and astrocytes in the affected spinal cord, *Slc31a1^+/^^−^* mice do not provide clear evidence for aberrant glial activation (Fig. 4). Thus, the lack of damaging glial activation coinciding with sustained motor function despite decreased motor neuron numbers in *Slc31a1^+/^^−^* mice suggests that motor function is determined not only by the number of motor neurons, but also by the physiological or pathological milieu in which they reside.

A homozygous missense variant in *SLC31A1* in humans (c.284G>A; p.R95H) has been identified as the cause of infantile seizures and profound neurodegeneration in monozygotic twins.^35^ These twins were reported to have survived past the age of 3 years. By contrast, a different homozygous missense variant (c.236T>C) that caused death at 1 month of age involved multisystem failure.^36^ Moreover, repeated miscarriages from the same consanguineous parents were speculated to have involved the same c.236T>C homozygosity.^36^ Homozygous knockout of *Slc31a1* in mice causes embryonic lethality.^16,17^ These findings collectively indicate that if mutations or deletions affecting *SLC31A1*/*Slc31a1* in humans/mice do not cause embryonic lethality, the functional impact on surviving individuals is characterized by neurological anomalies. This is in line with the canonical copper-related conditions of Menkes and Wilson diseases that arise due to mutations affecting the ATP-driven copper pumps ATP7A and ATP7B, respectively, and involve significant neurological complications.^37^ Furthermore, mutations affecting ATP7A are also associated with the neurological conditions of occipital horn syndrome and X-linked distal spinal muscular atrophy type 3.^38,39^ The propensity for mutations affecting copper handling mechanisms to result in neurological deficits is congruent with the requirement for copper in synaptic transmission and neuronal proteostasis,^40^ the natural slow turnover rate for copper within the CNS,^41^ the limited capacity of the CNS to respond to altered copper requirement,^8,9^ and selective sensitivity of the CNS to heterozygosity for functional Slc31a1^16,17^ (Fig. 1). It is also congruent with a study that showed the interactome of ATP7A immunopurified from neuroblastoma cells is enriched for gene products associated with nervous system diseases and mental disorders.^42^

In addition to neurological conditions in which abnormalities can be attributed to mutations affecting copper uptake and distribution pathways, evidence also exists to implicate altered copper availability in conditions not immediately associated with related mutations. For example, altered levels of copper are reported in affected brain regions from cases of schizophrenia,^43^ progressive supranuclear palsy,^44,45^ multiple sclerosis,^46^ Parkinson's disease,^45,47,48^ and Alzheimer's disease.^49–51^ Notably, these conditions are not necessarily characterized as diametrically opposed copper deficiency or accumulation. In Alzheimer's disease, for example, evidence for the accumulation of copper in extracellular amyloid plaques co-exists with evidence for intracellular copper deficiencies.^52^ Results such as these indicate that increased acquisition of copper in some anatomical and/or biochemical compartments of the CNS may occur at the expense of others, and that rather than supporting relatively simplistic copper delivery or copper chelation treatment strategies, promoting physiological redistribution of copper may be more beneficial.^53^

Herein, we compare *Slc31a1^+/^^−^* mice to *SOD1^G37R^* mice. *Slc31a1^+/^^−^* mice exhibit decreased spinal cord copper levels accompanied by a roughly proportional decrease in the number of α-motor neurons in the spinal cord but developed no overt molecular of physical disruptions. In contrast, although total spinal cord copper levels are reportedly unchanged or elevated in *SOD1^G37R^* mice,^7–9,54^ these mice exhibit a functional deficit in the activity of cuproenzymes, including SOD1, ceruloplasmin, and hephaestin.^7–9^ These changes are also apparent in the spinal cords of human ALS cases.^10^ Thus, there appears to be a disconnect between copper levels and copper-dependent processes in ALS. That pharmacologically or genetically enhancing spinal cord copper levels ameliorates the phenotype of these mice supports the notion that expression of mutant SOD1 induces a functional deficit in copper availability.^7–9^

The brain and spinal cord were the only tissues examined in the current study that exhibited altered copper levels in *Slc31a1^+/^^−^* mice, with no change observed in skeletal muscle, kidney, or liver (Fig. 1). This is consistent with previous findings for these mice.^16,17^ Functional Slc31a1 is required for copper acquisition by other tissues, as demonstrated by targeted deletion of *Slc31a1* from liver, resulting in hepatic copper depletion, albeit to an extent far less than commensurate with the ∼90% decrease in functional Slc31a1, indicating partial compensation.^55^ Cuproenzymes in the CNS, but not other tissues, are unsatiated for their requisite supply of copper in ALS model mice that overexpress transgenic SOD1, indicating that the CNS has a limited capacity to respond to increased demand for copper.^8,9^ Here, we show that this is associated with altered expression of numerous genes associated with copper handling (Fig. 3C and D). Based on these data collectively, it appears that in contrast to other tissues, the CNS is uniquely affected by disease-related changes in copper availability. However, results presented herein from the *Slc31a1^+/^^−^* mice show that a large decrease in CNS tissue copper content alone does not drive substantial functional changes that are present in mutant SOD1 models of ALS and human disease-affected tissue. This is evinced in the *Slc31a1^+/^^−^* mice by no change in motor function (Fig. 2D), limited changes in copper-related gene expression (Fig. 3A and B), and only a moderate decrease in SOD1 activity (Fig. 5B), despite the large decrease in tissue copper content (Fig. 1). This suggests that disturbances affecting intracellular distribution of copper and/or its accessibility to cuproenzymes may be a more important factor in neurodegenerative diseases such as ALS than tissue copper content alone. Phenotypic improvements achieved in mutant SOD1 mice through pharmacologically or genetically increasing spinal cord copper content^7–9^ may be effective by rectifying or bypassing these disturbances.

Overall, results presented herein support a clear relationship between tissue copper levels and motor neuron numbers in the spinal cord of *Slc31a1^+/^^−^* mice. The absence of overt signs of motor function impairment in *Slc31a1^+/^^−^* mice, however, suggests that the development of significant physical impairments in conditions such as ALS in which perturbations involving copper are implicated is not restricted to tissue copper content alone. Bioavailability of the copper is likely a more important consideration. Understanding how and why CNS copper bioavailability is affected will be essential to the development of associated therapeutic interventions. Importantly, delineating biochemical and/or anatomical sites of disrupted copper availability could likely be critical, with copper-related changes potentially affecting specific anatomical sites such as the synaptic cleft^56,57^ likely requiring distinct management relative to copper-related changes at other sites.

## Data Availability

The data underlying this article are available in the article.
